# Phocine Distemper Virus in Seals, East Coast, United States, 2006

**DOI:** 10.3201/eid1702.100190

**Published:** 2011-02

**Authors:** J.A. Philip Earle, Mary M. Melia, Nadine V. Doherty, Ole Nielsen, S. Louise Cosby

**Affiliations:** Author affiliations: Queen’s University Belfast, Belfast, UK (J.A.P. Earle, M.M. Melia, N.V. Doherty, S.L. Cosby);; Department of Fisheries and Oceans Canada, Winnipeg, Manitoba, Canada (O. Nielsen)

**Keywords:** Phocine distemper virus, morbilliviruses, epizootic, genomic sequence, phylogeny, central nervous system, persistent infection, mutations, United States, research

## Abstract

In 2006 and 2007, elevated numbers of deaths among seals, constituting an unusual mortality event, occurred off the coasts of Maine and Massachusetts, United States. We isolated a virus from seal tissue and confirmed it as phocine distemper virus (PDV). We compared the viral hemagglutinin, phosphoprotein, and fusion (F) and matrix (M) protein gene sequences with those of viruses from the 1988 and 2002 PDV epizootics. The virus showed highest similarity with a PDV 1988 Netherlands virus, which raises the possibility that the 2006 isolate from the United States might have emerged independently from 2002 PDVs and that multiple lineages of PDV might be circulating among enzootically infected North American seals. Evidence from comparison of sequences derived from different tissues suggested that mutations in the F and M genes occur in brain tissue that are not present in lung, liver, or blood, which suggests virus persistence in the central nervous system.

## Abstract 2

: Phocine Distemper Virus in Seals

In 1988, harbor seals (*Phoca vitulina*) and gray seals (*Halichoerus grypus*) died in large numbers off the coast of northern Europe (*1*). A virus was first isolated in April 1988, when widespread abortions and deaths among harbor seals were reported in the Kattegat area between Denmark and Sweden. The infection spread to the North, Wadden, and Baltic seas, killing 17,000–20,000 seals in northwestern Europe in 8 months. The virus subsequently was classified as a species of the genus *Morbillivirus* (family *Paramyxoviridae*) (*2**,**3*), *Phocine distemper virus* (PDV). The virus is believed to have originated in harp seals in which the infection is enzootic (*4*). Migrations of harp seals into the North Sea may have initiated the epizootic in harbor seals. Gray seals in the northeastern Atlantic Ocean also were infected, but disease was not as severe as in harbor seals (*5*).

A more recent outbreak occurred in Europe in 2002 (*6*). An estimated 30,000 harbor and gray seals died during this epizootic (*7**,**8*). The origin of this second epizootic 14 years after the first remains unknown. PDV may have jumped species into terrestrial carnivores, particularly mink, and reinfected seals (*9*), but this hypothesis remains unproven. Phylogenetic analysis of the hemagglutin (H) genes of PDV, together with those of other morbilliviruses, suggests that the reemergent 2002 PDV is more closely related to a putative recent ancestral PDV than to the 1988 isolates (*10*). Millions of seals of various species inhabit the waters surrounding North America; populations of most species are believed to be stable or increasing, and no epizootics on the scale of those reported in Europe have been reported. PDV disease in the United States was first reported in harbor seals on the east coast during the winter of 1991–92 (*11*), and serologic testing of gray and harbor seals suggested that a PDV-like strain or strains were circulating enzootically in the region (*12*). This circulation was attributed to an increased number of harbor seals (mainly immature animals) overwintering in southern New England (*13*). During the spring of 2006, deaths among seals (harbor, gray, and hooded) also increased along the coasts of Maine and Massachusetts. This increase was considered an unusual mortality event. Both dead and sick seals appeared nonemaciated. Live-stranded seals were weak and had generalized body tremors and spasms. Affected seals were taken to the Marine Science Education and Research Center (University of New England, Biddeford, ME, USA); investigations indicated that the pathologic changes were consistent with morbillivirus infection. Recent advances in virus isolation and genetic sequencing methods have provided us with better insight into PDV epizootiology in Europe and in North America.

## Materials and Methods

We isolated the 2006 virus from liver tissue of a harbor seal and confirmed it as PDV. To determine the phylogenetic relationship and possible origin of the isolate, we compared the virus RNA sequences and deduced amino acid sequences for the virus cell receptor attachment protein hemagglutinin (H) with those from various PDVs from both the 1988 and 2002 epizootics in Europe. We also investigated whether any differences in sequences between the PDV/USA2006 and the 2002 and 1988 viruses were likely to have occurred through sequencing errors, their tissue of origin, or adaption to Vero cells. Sequence information, when available for the phosphoprotein (P) membrane fusion (F), and internal matrix (M) protein genes also were compared for various viruses from outbreaks in Europe, the United States, and Canada during 1988–2006.

### Cells and Tissues

Vero and VeroDogSLAM (VDS) cells were grown in Dulbecco modified Eagle medium (Invitrogen, Carlsbad, CA, USA) supplemented with 5% fetal bovine serum. A blood sample from an infected seal from the 1988 epizootic was obtained from Albert Osterhaus, Erasmus University (Rotterdam, the Netherlands). Brain tissue from a harbor seal (designated PDV/3541UK) that was found off the coast of Scotland at the end of the 2002 epizootic and was PCR positive for PDV in the brain but not other tissues (lung, spleen, and lymph nodes) was obtained from Paul Jepson, Institute of Zoology, Zoological Society of London.

### Reverse Transcription–PCR and DNA Sequencing

Total RNA was extracted from infected cells and tissues by using TRIzol reagent (Invitrogen). cDNA synthesis was conducted by using oligo-dT primers and the SuperScript First-Strand Synthesis kit (Invitrogen). PCR was performed by using the High Fidelity Taq kit (Invitrogen). Morbillivirus universal P gene and β-actin primers (*14*) and further PDV primers to the H, F, M, and P genes designed to previously published PDV sequences are given in the Table (Table). DNA sequencing was performed by using a BigDye 3.1 Terminator Cycle sequencing kit (Applied Biosystems, Foster City, CA, USA) with primers listed in the Table. Completed PCR products were sent to the Genomics Core Facility, Queens University (Belfast, UK) for chromatographic preparation.

**Table:** Phocine distemper virus primers for reverse transcription–PCR and DNA sequencing*

Gene	Primer sequence, 5′ → 3′	Gene	Primer sequence, 5′ → 3′
P forward	ATGTTTATGATCACAGCGGT	F4 reverse	CCCGTAAACTTGGTCCAA
P reverse	ATTGGGTTGCACCACTTGTC	F5 forward	ATAATATAGGGTCACAGG
M forward	ATACTGCATTAACCCTGG	F5 reverse	CCTGTGACCCTATATTAT
M reverse	TTAGGTTGTTGGTCTTGGTAG	F6 forward	TTGTGCTTCTATCTTGTG
M1 forward	ATAACGATGATCTTGGCC	F6 reverse	CACAAGATAGAAGCACAA
M1 reverse	GGCCAAGATCATCGTTAT	F7 forward	GAATCCTCTGATCAAATC
M2 forward	ACACAGCTCAGAGATTCC	F7 reverse	GATTTGATCAGAGGATTC
M2 reverse	GGAATCTCTGAGCTGTGT	F8 forward	TGCAAGCTGGCACATCAG
M3 forward	ACTGGTGTTCGCCCTTGG	F8 reverse	CTGATGTGCCAGCTTGCA
M3 reverse	CCAAGGGCGAACACCAGT	H forward	CGAGGTTGAGGAAAGAAG
M4 forward	TTAAATTCCCAGTTCTTG	H reverse	CTCAATCTCGGTGGGTAC
M4 reverse	CAAGAACTGGGAATTTAA	H1 forward	AGGCAGTGCATCATCAAG
M5 forward	AGCCACTTGAATCTACGG	H1 reverse	CTTGATGATGCACTGCCT
M5 reverse	CCGTAGATTCAAGTGGCT	H2 forward	CAATCCTCTTGCTGACAC
F forward	GAGATTTGTGCACCTTTC	H2 reverse	GTGTCAGCAAGAGGATTG
F reverse	GC ATTGTTCTTGTAAAAGGC	H3 forward	AGATGGCTAGGTGATATG
F1 forward	TCATAGTCTCGATTCACC	H3 reverse	CATATCACCTAGCCATCT
F1 reverse	GGTGAATCGAGACTATGA	H4 forward	CACCGGGGTTTCATAAAG
F2 forward	TTATCAACAATTGGAATC	H4 reverse	CTTTATGAAACCCCGGTG
F2 reverse	GATTCCAATTGTTGATAA	H5 forward	GGATTATTATGAGGGTAC
F3 forward	TGCAGGTGCAGCTCTAGG	H5 reverse	CAATAGCATGATCACTCC
F3 reverse	CCTAGAGCTGCACCTGCA	H6 forward	GGAGTGATCATGCTATTG
F4 forward	TTGGACCAAGTTTACGGG	H6 reverse	GTACCCTCATAATAATCC

*H, hemagglutinin; P, phosphoprotein; F, fusion; M, matrix.

## Results

### Isolation and Identification of USA 2006 as PDV

**Figure 1:**
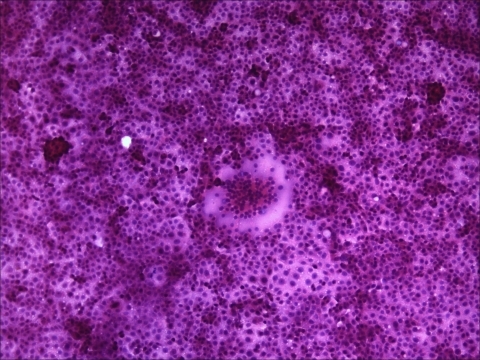
VeroDogSLAM cells 30 h postinfection inoculated with tissue (liver) homogenate from a dead adult female harbor seal from Maine that died in July 2006. Syncytia are seen in the monolayer, which is stained with hematoxylin and eosin (original magnification ×100).

Signaling lymphocyte activation molecule (SLAM) is a receptor for both vaccine and wild-type strains of measles virus and for canine distemper and rinderpest morbilliviruses (*15**,**16*). VDS cells have been used successfully to isolate PDV from experimentally infected ferrets (*Mustela putorius furo*) (*17*), and we recently confirmed that this molecule also is used as a receptor for PDV (M. Melia et al., unpub. data). The USA 2006 virus was isolated by inoculating homogenized liver tissue from a harbor seal onto VDS cells, which resulted in syncytia formation (Figure 1). Reverse transcription–PCR (RT-PCR) was initially conducted by using previously published morbillivirus universal P gene primers (*14*). The PCR product was sequenced and aligned with morbillivirus sequences for this target region and showed 100% homology with PDV (data not shown).

### Origin of Sequence Data

Selected gene sequences from the PDV/Ulster 88, PDV/NL88, PDV/DK 88, and PDV/DK2002 viruses from Europe were available for comparison with the PDV/USA2006 strain (*10**,**18**–**21*). In addition, we included partial P gene sequences obtained from tissues and nasal swabs of northern seas otters (*Enhydra lutris*) from an unusual mortality event in south-central Alaska in 2006, as well as samples from a harp seal found in the Gulf of St. Lawrence (Canada) in 1991 and from a hooded seal on the New Jersey, USA, coast in 1998 (*22**,**23*).

Because of improvements in techniques of sequence determination from those available in 1988, we initially resequenced the PDV/Ulster88 virus H, M, and F genes. We have designated this “new” sequence PDV/Ulster88n. For the same reason, and to determine whether adaptation to Vero cells changed the virus sequence, particularly in the cell receptor attachment H protein, we obtained RNA directly from a blood sample used to isolate the PDV/NL88 strain and conducted RT-PCR directly. We designated this “new” sequence for the H, F, and M genes as PDV/NL88n. The sequence obtained from the central nervous system tissue of the seal from the 2002 epizootic is designated PDV/3541UK.

### Nucleotide and Deduced Amino Acid Sequence Comparison

Previous studies have shown that the H protein is the most variable and that comparison of the PDV/2002 and PDV88 sequences from Europe showed 8 minor amino acid changes (*10**,**21*). The sequences for the PDV/Ulster88 and PDV/NL88 viruses had been obtained from isolates in Vero (African green monkey kidney) cells, unlike the 2002 sequences, which were obtained directly from lung tissue. To determine whether errors may have occurred in the PDV/Ulster88 sequence, we initially compared the original PDV/Ulster88 and new PDV/Ulster88n H gene sequences. Changes in PDV/Ulster88 compared with the consensus sequence for all the viruses at bases 910, 911, 1134, and 1135 are not mirrored in the PDV/Ulster88n sequence, which indicates that these are likely to have resulted from sequencing errors and therefore do not reflect real differences in the more recent isolates. Similarly, to determine whether changes in the original PDV/NL88 strain are likely to have resulted from adaption to Vero cells, we compared the original PDV/NL88 H sequence with PDV/NL88n, which was amplified directly from a blood sample. Differences of PDV/NL88 from the consensus at bases 23 and 1711 are not reflected in the PDV/NL88n sequence, which indicates that these are likely to have resulted from tissue culture adaptation and/or sequencing errors.

**Figure 2:**
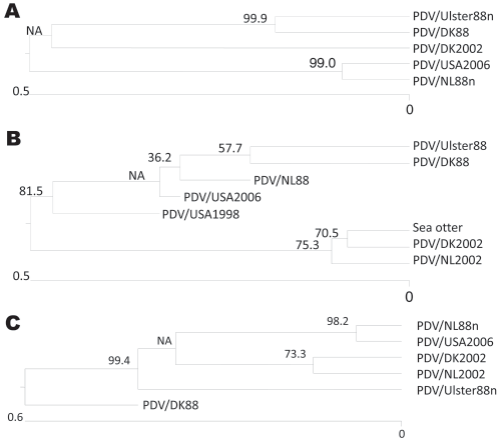
Phylogenic relationship of PDV/USA2006 to viruses from the 1988 and 2002 epizootics in Europe based on hemagglutinin (H) and phosphoprotein (P) gene sequences. A) Phocine distemper virus (PDV) H gene sequences used for the alignments were from PDV/DK2002 (lung), GenBank accession no. FJ648456; PDV/DK88 (isolated in Vero cells), GenBank accession no. Z36979; PDV/Ulster88n (isolated in Vero cells), PDV/NL88n (blood), GenBank accession no. D10371 (minus described changes in this study); and PDV/USA2006 (isolated in VeroDogSLAM cells), GenBank accession no. 1375698. B) PDV P gene sequences used for the alignments were from PDV/DK2002 (lung), GenBank accession no. –af52587; PDV/NL2002 (lung) GenBank accession no. af52588; PDV/DK88 (isolated in Vero cells), GenBank accession no. –x75960; PDV/Ulster88 (isolated in Vero cells), GenBank accession no. D10371; PDV/NL88 (isolated in Vero cells), GenBank accession no. af525289; PDV/USA1998, GenBank accession no. ay3323389; and PDV/USA2006 (isolated in VeroDogSLAM cells), GenBank accession no. 1375683. Unrooted neighbor-joining phylogenetic trees in A and B were constructed by using the MegAlign version 7.1 package (DNASTAR, www.dnastar.com) with the ClustalW method (www.clustal.org). C) Unrooted neighbor-joining phylogenetic tree for concatenated H and P squences constructed by using ClustalV. Scale bars denote number of nucleotide substitutions per site along the branches. Percentage bootstrap values, indicating the significance of clusters, are shown.

The newly isolated PDV/USA2006 virus has 11 aa changes in the H gene (GenBank accession no. 1375698), compared with the PDV/DK2002 strain at codons 176, 200, 218, 221, 276, 327, 399, 432, 561, 561, and 576. However, the sequences at codons 200, 218, 276, 399, and 432 are in common with the PDV/NL88n strain. Similarly, silent mutations at nt 180, 564, and 1728 are the same as PDV/NL88n. With the exception of codon 564, all other differences of PDV/USA2006, compared with PDV/DK2002, reflect the consensus sequence. All of these observations suggest that the similarities of PDV/USA2006 to the 1988 virus may be due to circulation of multiple lineages. A phylogenic analysis for the H gene is shown in Figure 2, panel A.

**Figure 3:**
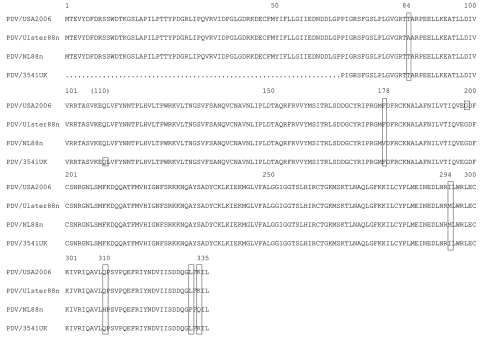
Amino acid alignment of the matrix protein of phocine distemper virus (PDV) strains PDV/USA2006, PDV/Ulster88n, PDV/NL88n, and PDV/UK3541. The first 233 nt of the matrix gene of PDV/3541UK, as indicated by a dotted line, are undetermined. Large boxes indicate amino acid changes and small boxes the position of silent mutations.

**Figure 4:**
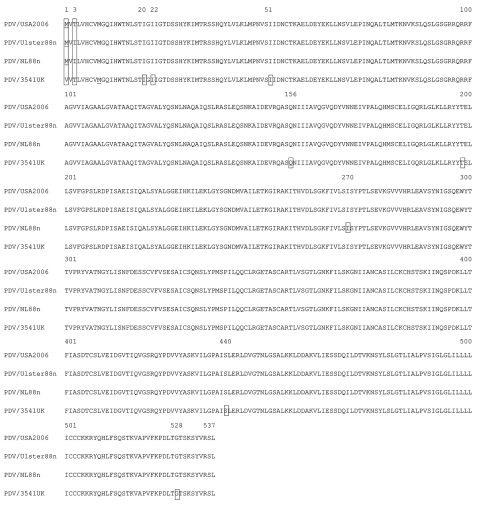
Amino acid alignment of the fusion protein of phocine distemper virus (PDV) strains PDV/USA2006, PDV/Ulster88n, PDV/NL88n, and PDV/UK3541. Large boxes indicate amino acid changes and small boxes the position of silent mutations.

Few amino acid changes are found in the P (GenBank accession no. 1375683) and M (Figure 3) genes of PDV/USA2006, compared with the other viruses. Complete P gene sequences were available for the DK2002, NL2002, Ulster, NL and DK 88 viruses. Partial sequences from the 1991 Canadian and 1998 USA viruses, as well as the sea otter 2006 virus, also were used for comparison (*22**,**23*). The USA/2006 P gene differs from the more recent 2002 viruses at codon 152 but is the same as all three 1988 isolates. Other minor variations between strains at the sequence level align PDV/USA2006 most closely to PDV/NL88. A phylogenic analysis based on the P gene is shown in Figure 2, panel B, and a concatenated tree based on both the H and P genes (for viruses where both sequences are available) is shown in Figure 2, panel C. The M and F gene sequence of PDV/USA2006 were compared with the sequences of PDV/Ulster88n, PDV/NL88n, PDV/DK88, and PDV/3541UK. The PDV/USA2006 M and F protein amino acid sequences (Figures 3, 4) reflect at least 1 of the other viruses at all positions. PDV/Ulster 88 M sequence differs from the other viruses at codon 84, whereas PDV/NL88n differs at codons 178, 310, 331, and 333. The first 233 nt of the PDV/3541UK M gene could not be amplified. However, alignment of the remaining sequence showed amino acids to be in common with >1 of the other viruses. In the F gene, an amino acid change occurred in the initiation codon from Met to Val in PDV/3541UK, compared with the other viruses, and silent mutations occurred at codons 20, 22, 51, 156, 198, 440, and 528.

## Discussion

We confirmed that at least some of the deaths in seals that occurred around Maine and Massachusetts in 2006 resulted from PDV infection and conducted sequence alignments with other strains derived during 1988–2006. Müller et al. reported 8 aa changes in the H gene between the 1988 and 2002 European strains of PDV (*21*). We have shown that 4 base changes in PDV/Ulster88, compared with the 2002 isolates, probably resulted from errors by resequencing this virus (PDV/Ulster88n) by using current technology. Furthermore, the PDV/NL88 strain when reisolated directly from a blood sample (PDV/NL88n) also showed fewer differences to 2002 strains, which in this case may have been due to adaptation to Vero cells, although sequencing errors cannot be ruled out. Phylogenetic analysis of the individual H and P gene sequences, as well as combined concatonated sequences (Figure 2), suggests that PDV/USA2006 is more closely related to PDV/NL88n than to the 2002 European viruses. Although sequence information was limited, we found that the 2006 sea otter virus P gene sequence was identical to that of the PDV/DK2002 virus. Therefore, the two 2006 viruses, 1 each from the US Atlantic and Pacific coasts, might have had different origins, but this possibility requires further investigation. The limited sequence information for the P gene of the 1991 virus from Canada and the 1998 virus from the United States is identical to that of the P gene of PDV/DK2002. More sequence information for these North American viruses, particularly for the H gene, might give further insight into their origin and evolution.

Unlike the 2002 viruses, from which RNA sequences were obtained directly from tissues, the PDV/USA2006 strain was isolated into VDS cells, which might explain the differences, particularly in the H gene. Sequencing viruses isolated in these cells is preferable to sequencing them isolated in Vero cells because of expression of the SLAM receptor. The SLAM binding site of the H protein has been shown to be conserved across all known morbilliviruses (*24*). We found that the residues were conserved in all of the PDVs tested, including the 1988 strains that had been isolated in Vero cells. Nielsen et al. (*10*) suggested that the higher identity of the H protein in 1988 strains than in PDV/DK2002 could have been due to passaging of the former in Vero cells. By comparing directly amplified PDV/NL88n sequences from a blood sample, we demonstrated that the similarities with the USA/2006 isolate are unlikely to have resulted from tissue culture adaption of the latter.

The M and F amino acid sequences are in common with >1 of the other viruses. The first 233 nt of the M gene of PDV/3541UK could not be amplified despite successful amplification of the F gene and the housekeeping mRNA β-actin. Therefore, mutations may remain to be identified, and those mutations may account for the lack of primer specificity in this region.

A total of 8 mutations were found in the PDV/3541UK F sequence, compared with those of PDV/USA2006, PDV/Ulster88n, and PDV/NL88n. Seven of these changes were silent. The substitution of Met to Val in the first codon may be particularly noteworthy. Perhaps, in PDV/3541UK, GUG can be used as the initiation codon or initiation may take place at the next available Met. In the latter case, a fully functional F protein would not be produced in the brain of this animal. Truncations, mutations, and deletions in the cytoplasmic domain of the F protein occur in the persistent measles central nervous system complications (subacute sclerosing panencephalitis and measles inclusion body encephalitis) (*25*). Sequencing of more F genes from seal brain tissue is necessary to determine whether the observed substitutions are a common feature and whether they are associated with persistent infection.

In this study we confirmed that the virus isolated from the US 2006 unusual mortality event in seals was PDV. The similarity of this isolate to the PDV/NL88n virus suggests that PDV/USA2006 may have reemerged independently of the 2002 PDVs and that multiple lineages of PDV may be circulating enzootically among the large populations of North American seals. Multiple lineages of canine distemper virus occur worldwide and affect different carnivore species (*26*). The Maine 2006 unusual mortality event in seals never progressed to a full-blown epizootic as occurred in Europe, perhaps because of differences in the pathogenicity of the US 2006 virus itself, or more likely, because several PDV viruses are circulating among populations of seals that are large enough to maintain them enzootically without large-scale die-offs. Contributing to the early detection of PDV in the affected seals were range extension and rapidly increasing populations of harbor and gray seals (*13*) that may have had a high prevalence of immunologically naive individuals, coupled with an active surveillance program to rehabilitate stranded seals. The findings of increased base substitutions in the F gene and lack of an amplifiable product at the start of the M gene from brain tissue of an animal in 2002, compared with those obtained from lung, liver, or blood, raises the possibility that mutations could occur in the central nervous system and might be associated with persistent infection.
